# Recruitment Barriers and Potential Strategies for Inclusion of Older Asian Americans in Alzheimer's Disease Research

**DOI:** 10.1177/00914150241253253

**Published:** 2024-05-16

**Authors:** Alva Hồng Anh Nguyễn, Guerry M. Peavy, Namkhuê Võ, Sheri Thompson

**Affiliations:** 1 Shiley-Marcos Alzheimer's Disease Research Center, 8784 University of California San Diego, La Jolla, CA, USA; 2 Department of Neurosciences, 8784 University of California San Diego, La Jolla, CA, USA; 3 Alzheimer's Disease Cooperative Study, 8784 University of California San Diego, La Jolla, CA, USA; 4 MADURA Undergraduate Research Program, School of Public Health and Human Longevity, 8784 University of California San Diego, La Jolla, CA, USA

**Keywords:** Alzheimer's disease, Asian Americans, recruitment, research barriers, dementia, advancing diversity in aging research

## Abstract

The makeup of the US population of older adults continues to become more diverse as numbers from ethnic subgroups increase. However, these subgroups are generally underrepresented in research focused on Alzheimer's disease and related dementias (ADRD). This paper examines barriers to recruitment for older Asian Americans, to underpin potential strategies for future research, with particular emphasis on recruitment of Vietnamese Americans. The paper discusses three recommended strategies: implementing appropriate recruitment outreach methods, establishing and maintaining community partnerships, and adopting flexible and convenient assessment methods. All three complementary approaches may be applied to improve Vietnamese American aging research participation. This has the potential to promote early intervention, foster longevity, ameliorate health disparities, and reduce healthcare burdens for this population.

Dementia, broadly defined as a group of symptoms that includes impaired cognition and difficulty performing daily activities independently, is a major public health problem. The most common cause of dementia is Alzheimer's disease (AD), a neurodegenerative process that accounts for an estimated 50–70% of total dementia cases (Zhang et al., 2021). It is known that a combination of genetic and environmental factors contributes to the development of AD. Depending upon the stage of disease progression, symptoms may include forgetting recent conversations, getting lost in familiar surroundings, inability to perform routine activities, poor judgment, and changes in mood and personality.

Life-extending medical advances in developed countries have resulted in increased AD prevalence. According to a special report by the Alzheimer's Association (Alzheimer's Association, 2023), approximately 6.7 million people in the US are living with Alzheimer's disease and related dementias (ADRD). In addition to the debilitating impact of AD on the lives of patients and families, annual healthcare costs for ADRD in the US in 2023 was an estimated $355 billion, excluding unpaid caregiving (Alzheimer's Association, 2023). Increasing costs over time are likely to further exacerbate health disparities experienced by minority and lower socioeconomic status individuals.

Asian Americans include individuals from more than 20 countries in Eastern, Southern and Southeastern Asia, and the Indian subcontinent (Budiman & Ruiz, 2021), with descent from groups that, among others, include Chinese, Filipino, Indian, Japanese, Korean, and Vietnamese. Asian Americans are anticipated to become the largest immigrant group in the United States by 2055 (Budiman & Ruiz, 2021). It is estimated that the number of Asian Americans and Pacific Islanders (AAPIs) over the age of 64 will increase by an estimated 352% by 2060 (National Asian Pacific Center on Aging, 2021).

Here we address barriers to healthcare in the Asian American population, highlighting obstacles to dementia research participation. We present a review of strategies for research recruitment in older adults in the Asian American population.

Health disparities are defined by the U.S. Center for Disease and Control Prevention (CDC) as “preventable differences in the burden of disease, injury, violence, or opportunities to achieve optimal health that are experienced by socially disadvantaged populations” (Center for Disease and Control Prevention [CDC], 2023). Asian Americans face barriers and disadvantages that impact a variety of circumstances that determine health outcomes. It is estimated that more than 62% of Asian Americans have limited English proficiency. They are twice as likely to lack a healthcare provider and to have unmet medical needs compared to those with good English proficiency (Jang & Kim, 2019). They are less likely to attend annual health check-ups and to have health insurance coverage (Jang & Kim, 2019). Yet, as with other populations, particularly with aging, the risk of developing AD and related dementias faced by Asian Americans underscores the importance of their inclusion in ADRD research. This research may yield insights into policies which could reduce financial burden on those immigrant households that are least equipped to provide caregiving when faced with the development of AD in a family member. Finally, such research carries the potential to increase understanding of genetic and non-genetic risk factors for ADRD that may differ among various subgroups of the Asian American population.

As with other AAPI groups, the Vietnamese population is adversely impacted by a lack of research representation. With a 40% increase from 2000 to 2020, the Vietnamese population is now the fourth largest Asian subgroup in the United States, numbering over two million people (Lee & Cassado, 2019). Yet research studies addressing aging and AD in this population are limited. Among the 2,505 Vietnamese participants in a recent study by Park et al. (2023), 65% spoke only Vietnamese. Vietnamese Americans comprised their largest AAPI subgroup of non-English speakers.

Limited English proficiency often leads to problems utilizing healthcare services and is a major obstacle to representation of Asian Americans in aging research. Language barriers not only affect access to healthcare services but also affect the ability to obtain information (e.g., facts about AD) and to understand the importance of participating in aging research. To address this issue, research studies have developed implementation approaches (e.g., providing interpreter service, carefully translating materials) (Jang & Kim, 2019) specifically tailored for Asian Americans with limited English skills.

The National Alzheimer's Coordinating Center (NACC) reported that AAPIs only accounted for 2.6% of participants in the ADRD studies from a total of 44,359 participants (National Alzheimer's Coordinating Center [NACC], 2021). In addition, clinical research involving AAPIs has received less than 1% of federal funding in the past 10 years, and the Asian and underrepresented minority investigators in behavioral and social sciences tend to encounter more struggles with obtaining government funding than White investigators (Dong, 2019). The gap in research efforts is illustrated by the fact that Asian investigators are less likely to receive funding from the National Institute of Health (NIH) and National Science Foundation (NSF) than White investigators (Dong, 2019). Between 1992 and 2018, only 0.17% of projects funded by NIH were focused on health issues of Asian Americans, Native Hawaiian, and Pacific Islanders (Dong, 2019). In addition, Asian Americans are treated as a homogeneous group, with consequent loss of knowledge concerning differences in these subgroups. Moreover, according to the NIH and the NSF, Asian Americans are not considered underrepresented minorities, limiting access to funds earmarked for underrepresented minority research (Dong, 2019).

Findings from multiple studies suggest three particularly important strategies for research recruitment of older adults in the Asian American population: (1) implementing appropriate recruitment outreach methods, (2) establishing and maintaining community partnerships, and (3) adopting flexible and convenient assessment methods (Mukherjea et al., 2018; Park et al., 2021, 2023).

The first strategy involves the implementation of appropriate recruitment outreach methods. In addition to educating participants, recruitment-focused outreach events are designed to promote interest in ADRD research and involve family members in a variety of roles (e.g., initiating follow-up contacts with research personnel, scheduling appointments, serving as research study partners) to enhance engagement and recruitment. These outreach plans must be culturally and linguistically appropriate for the targeted subgroups of Asian Americans with distinct cultures, languages, and customs. Researchers should tailor information messaging, and channels for the target group of interest that includes both potential participants and their family members.

In addition to personnel who can communicate in the participant's native language, tailored digital and print media can be used to deliver information at low cost. Research developing the CARE Registry found that different AAPI subgroups tended to use different social media channels (Park et al., 2023). Social media is useful in supplementing dissemination of information to the targeted group and/or through community leaders and participants. Finding social media platforms most often used by Vietnamese Americans should be beneficial for spreading information within the community.

While passive recruitment methods such as social media postings, flyers, emailed newsletters and web links are useful in many ways, studies have found that they are limited in their ability to recruit Asian Americans (Mukherjea et al., 2018; Park et al., 2023), noting that recruiting participants through community organizations may be more effective. The CARE study reported that 69.2% of its older adult participants engaged in CARE as a result of community recruitment efforts, while only 10% were referred by word-of-mouth and 8% through general social media advertisements (Facebook and other media posts) or websites (Park et al., 2023). Activities at festivals, community centers, and cultural and faith-based events, where target populations tend to congregate, are likely to be most beneficial. Community outreach events that aim to educate residents about AD and dementia can build trust between the research team and potential research participants.

The second strategy critical to recruitment success is establishing community-centered partnerships. Collaborating with community and faith leaders from target groups can help to bridge the gaps between the dominant culture and subsets of the population that have led to long-standing health disparities, including disparities in research access and participation. The effectiveness of recruitment outreach activities will be limited, without collaboration and approval of long-established local community leaders and organizations (Mukherjea et al., 2018). A study by Mukherjea et al. (2018) serves as an example. Working with community gatekeepers to recruit South Asian participants into tobacco use research in Northern California, this study noted that women who commit to Sikh faith were prohibited from discussing tobacco use with researchers until a *community leader* endorsed study participation. Similarly, the CARE registry study (Park et al., 2023) succeeded in recruiting more than 7,000 older AAPI participants within a year using online meetings with diverse partners to introduce the study. In general, community leaders and acquaintances may be enlisted as partners to increase awareness across the community, correct misconceptions, and secure trust in research programs.

The third strategy for encouraging recruitment of Asian Americans to research is identifying and adopting flexible and convenient screening and assessment methods. With the Covid-19 pandemic, reliance on remote screenings and assessments gained popularity among healthcare researchers. Indeed, many studies could only continue during the pandemic when digital formats were adopted. Additional advantages of remote recruitment and assessment formats continue to expand outreach to those with limited transportation resources and other barriers to in-person visits. The decision of the CARE Registry (Park et al., 2023) to conduct their research using both in-person and online tools during the pandemic is informative. While 69 CARE participants completed a survey online, 54 completed a paper survey (Park et al., 2021). CARE staff also administered surveys during outreach activities via phone calls and zoom meetings in the participants’ preferred language. During the pandemic, about 77% of the Registry's recruitment events were held online, with the rest held in-person at community organizations and gathering places (churches, pagodas) (Park et al., 2023). These experiences suggest that remote recruitment efforts can be an asset, particularly when combined with community partnerships and in-person engagement with the target population.

Despite some of the benefits of digital technologies, Park et al. (2021) recommend offering in person research screening options for older people with limited English proficiency and for those who are not familiar with social media and digital technologies. Forty-four percent of their focus group participants opted to complete paper surveys in person, citing a lack of internet access and trouble interpreting online survey instructions and response formats. Some participants noted preferences for one-on-one meetings with research staff to clarify instructions and procedures. The process of working closely with and monitoring study participants throughout screenings and other assessments has the potential to reinforce motivation, commitment, and interest in study participation.

## Discussion

The critical need for inclusive and effective strategies to engage and recruit older Asian American adults into ADRD research is of utmost importance, given the predicted growth of this segment of the population and the pressing demand for research that addresses specific needs. However, the underrepresentation of Asian Americans in ADRD research presents a significant challenge. Over the past decade, concerted efforts have been made by organizations and researchers to address this issue, focusing on strategies that aim to boost recruitment rates among older Asian Americans. The Asian Cohort for Alzheimer's Disease (ACAD) pilot study has shown that it is feasible to recruit Asian Americans and Canadians in a study that includes Chinese, Korean, and Vietnamese participants with a goal of understanding genetic and lifestyle risk factors for AD (Ho et al., 2024).

The proposed strategies outlined in this manuscript are designed to work together to enhance participant recruitment. Culturally and linguistically appropriate research outreach and community partnerships are two of the most vital strategies for improving recruitment rates among older Asian Americans. Establishing a collaborative multicultural/multilingual research team capable of effective communication with participants and community partners in their preferred language is also crucial. This includes careful translations of all materials, interpreting information about consenting and procedures, and providing ongoing culturally sensitive interactions in the recruitment and assessment process. The support of a bilingual research team to communicate with individuals in their preferred language is critical to optimally confronting barriers such as problems engaging, confusion, shame, and a lack of trust in research personnel Table 1.

**Table 1.** Barriers and Potential Strategies for Recruitment of Asian Americans.

Problems	Strategies
Limited English proficiency	Vietnamese-speaking research staff: - Actively leading recruitment and assessment activities - Engaging in accurately translating all research materials
Mistrust of research investigators	Coordinating with community gatekeepers to request help in communicating the potential benefits of the research study
Lack of knowledge and /or misconceptions about AD	Offering local community education about Alzheimer's disease (AD) in the language of potential participants Destigmatizing AD as a medical condition, offering education on the importance and value of early detection and potential interventions
Shame, embarrassment, isolation associated with AD diagnosis	Promoting family emotional and practical support to decrease participant isolation and increase empathy Educating about AD as a medical condition
Technological limitations	Offering screening by phone for participants not comfortable with online communications
Lack of family assistance with transportation and/or screening completion	Providing direct assistance to participants by bilingual research staff, in person, online, or by phone
Low motivation for research participation	Emphasizing personally meaningful potential benefits, such as contributing intellectual value to future generations, being a community representative, and expanding knowledge about aging and AD in underrepresented populations Encouraging family or friends to assist individuals with AD to gain understanding about the disease Offering incentives such as transportation vouchers, gift cards, complimentary wellness sessions

Community partnerships are also expected to play a vital role in recruitment success. Establishing strong and ongoing relationships with community gatekeepers has been shown to be a powerful ally in establishing trust with potential research participants and their families. Internal supporters who fully appreciate the culture and customs of the community can help to bridge the distance between researchers and Asian American community members. Involvement of respected community leaders will contribute to the success of research recruitment.

In addition, maintaining flexibility in online and in-person assessment methods is essential to accommodate participants’ preferences. While in-person assessments may be preferable, online methods offer alternatives for older adults who do not have reliable transportation options. Study protocols can be adapted based on participants’ convenience and researcher resources, including screening through phone or video and using mail-in bio-samples (e.g., saliva kits) to facilitate participation.

While addressing language proficiency and trust in research personnel can reduce some barriers, health literacy remains one of the biggest challenges in research recruitment of underrepresented groups. Health literacy can be enhanced by fostering strategic partnerships with community gatekeepers who can help disseminate essential knowledge and support research implementation. Elevating health literacy is key to advancing early detection and intervention, particularly within minority and ethnic groups. Educating older Vietnamese American communities about ADRD is vital to reducing dementia-related stigma and burden on families, by encouraging initiation of professional support.

To effectively address hardships resulting from healthcare disparities including those associated with limited knowledge about ADRD risk factors in Asian Americans, national efforts should concentrate on five objectives: enhancing population health literacy; overcoming language barriers, cultural sensitivities and misconceptions; promoting early detection of cognitive impairment; providing interventions to address racial/ethnic disparities; and managing financial burdens associated with healthcare. Addressing the unique lifestyles, cultures, and communication methods of Asian subgroups poses a significant recruitment challenge. Overcoming linguistic obstacles, sharing knowledge about ADRD, and conveying tailored messages are strategies that can motivate older Asian Americans to participate in studies. Researchers must employ creativity, adaptability, and evaluative approaches to identify the most effective methods for engaging Asian Americans in research.

Often overlooked in the past, the role of the study partner (also referred to as informant, informal caregiver) has more recently emerged as a crucial aspect of ADRD research (Elliott, 2020; Peavy, 2023). Agreement to participation in research, participation itself, and adherence to study procedures are often enhanced by the contributions of the study partner, who may be a family member or friend. This involvement provides clear benefits such as assistance with logistical support and enhancing social engagement. Further, information from study partners concerning the participant's cognitive and functional abilities supports accurate diagnosis and tailoring of support services. The progressive nature of ADRD makes the study partner's role indispensable for capturing nuanced changes in the participant's condition.

## Additional Resources

To confront health disparities in healthcare access and education, the National Asian Pacific Center on Aging (NAPCA), a non-profit organization, has been instrumental in providing resources to enhance the ability of Asian Americans with limited English proficiency to obtain equitable access to federal health services (Schafer et al., 2017). NAPCA's initiative is pivotal in improving the quality of life and well-being for Asian Americans. Promoting diversity in research participation is a significant step toward bolstering trust and engagement among underrepresented groups.

The World Health Organization (WHO) has underscored dementia as a critical public health concern and prioritized increasing public awareness. Initiatives to conduct health education sessions for Asian immigrants aim to enhance mental health literacy and address the underrepresentation of minorities in research (Lee & Casado, 2019). These efforts are expected to lead to more equitable research and health interventions with the goal of improving cognitive health and overall quality of life for older Asian immigrants.

Although well-established healthcare organizations like NAPCA and WHO have developed programs to raise awareness and support minority groups, there is a need for localized initiatives to develop social and cultural interventions for ADRD. Alzheimer's Disease Research Centers (ADRCs) across the US are funded by the National Institute on Aging to address a wide variety of efforts, including developing strategies for recruitment and retention of older adults in research, fostering the inclusion of caregivers, providing services for participants, families and community members, strengthening dementia education of students at all levels, and including primary care providers in research endeavors. Finally, ADRCs are in a unique position to encourage inclusion and diversity in research staff and trainees, as well as research participants, family members and community partners (Elliott, 2020). These centers must continue to promote research participation by underrepresented groups to discover and highlight disease complexities within these populations, that include Asian Americans.
